# Differential Gene Expression Profiles and Selected Cytokine Protein Analysis of Mediastinal Lymph Nodes of Horses with Chronic Recurrent Airway Obstruction (RAO) Support an Interleukin-17 Immune Response

**DOI:** 10.1371/journal.pone.0142622

**Published:** 2015-11-12

**Authors:** Alexandra Korn, Donald Miller, Lynn Dong, Elizabeth Louise Buckles, Bettina Wagner, Dorothy Marie Ainsworth

**Affiliations:** 1 Department of Clinical Services, College of Veterinary Medicine, Cornell University, Ithaca, New York, United States of America; 2 Department of Microbiology and Immunology, College of Veterinary Medicine, Cornell University, Ithaca, New York, United States of America; 3 Department of Biomedical Sciences, College of Veterinary Medicine, Cornell University, Ithaca, New York, United States of America; 4 Department of Population Medicine and Diagnostic Sciences, College of Veterinary Medicine, Cornell University, Ithaca, New York, United States of America; Virginia Tech University, UNITED STATES

## Abstract

Recurrent airway obstruction (RAO) is a pulmonary inflammatory condition that afflicts certain mature horses exposed to organic dust particulates in hay. Its clinical and pathological features, manifested by reversible bronchoconstriction, excessive mucus production and airway neutrophilia, resemble the pulmonary alterations that occur in agricultural workers with occupational asthma. The immunological basis of RAO remains uncertain although its chronicity, its localization to a mucosal surface and its domination by a neutrophilic, non-septic inflammatory response, suggest involvement of Interleukin-17 (IL-17). We examined global gene expression profiles in mediastinal (pulmonary-draining) lymph nodes isolated from RAO-affected and control horses. Differential expression of > 200 genes, coupled with network analysis, supports an IL-17 response centered about NF-κB. Immunohistochemical analysis of mediastinal lymph node sections demonstrated increased IL-17 staining intensity in diseased horses. This result, along with the finding of increased IL-17 concentrations in lymph node homogenates from RAO-affected horses (P = 0.1) and a down-regulation of IL-4 gene and protein expression, provides additional evidence of the involvement of IL-17 in the chronic stages of RAO. Additional investigations are needed to ascertain the cellular source of IL-17 in this equine model of occupational asthma. Understanding the immunopathogenesis of this disorder likely will enhance the development of therapeutic interventions beneficial to human and animal pulmonary health.

## Introduction

Certain mature horses, when exposed to hay particulates and stable dust, spontaneously develop a pulmonary disorder called recurrent airway obstruction (RAO). This disease, characterized by reversible airflow obstruction, bronchial hyper-responsiveness, excessive mucus production and airway neutrophilia [1] shares many of the clinical and pathological features of occupational asthma in humans [2]. This similarity, coupled with the finding that horses have more similar life spans and lung anatomy to humans than do mice, makes the equine species an attractive animal model for investigations of human asthma [3].

Like asthma, RAO may be driven by an excessive innate immune response as well as by specific T-helper lymphocyte-mediated reactions [4]. No consensus has yet been reached as to the immunological basis of RAO as some studies support a Th-2 basis [5] while others fail to support a polarized immune response [6,7]. Interestingly, cytokine profiles of bronchoalveolar lavage fluid (BALF) cells obtained from horses with chronic RAO exhibit increased gene expression of IL-17 [8,9,10]. However, the primary cellular source of equine IL-17A mRNA (granulocytes, NK cells, CD4^+^, CD8^+^, innate lymphoid cells) remains to be ascertained [11]. Until recently, most of the investigations into the immunopathogenesis of RAO have evaluated the expression of a limited number of genes in cells of the peripheral blood, BALF or airway biopsies using kinetic PCR. Yet, in studies of multi-factorial diseases like asthma, high throughput gene expression technologies such as microarrays offer the advantage of identifying whole families of differentially expressed genes and related pathways. As a result, hypotheses regarding the genes responsible for certain disease phenotypes can more easily be generated and tested [12].

Because immunological responses in the lung are reflected in the molecular events in the immune cells of the draining lymph nodes [13,14], we examined the global gene expression of cells in the draining (mediastinal) lymph nodes obtained from horses chronically-affected with RAO and non-affected control horses. We hypothesized that the gene expression profiles, immunohistochemical staining of the lymph node tissue, and analysis of lymph node homogenates would support an IL-17 dominated immune response in horses with chronic, active RAO. We provide data in strong support of this hypothesis.

## Materials and Methods

### Experimental design

#### Animals

Fourteen adult horses, 5 mares and 9 geldings (450–575 kg) were studied. Seven principal horses (mean ± SD, 18.7 ± 3.5 years of age) had a history of developing RAO when stabled and exposed to dusty hay (natural challenge environment, NCE). Seven control horses (13.9 ± 4.0 years of age), free of respiratory diseases for the past 8 years, had a history of remaining healthy when exposed to the NCE. The criteria used to define RAO were the development of an accentuated breathing effort; a maximum pleural pressure change from peak inspiration to peak expiration (ΔPpl_max_) ≥15 cm H_2_O; and pulmonary neutrophilia characterized by ≥ 25% of the total nucleated cells in the bronchoalveolar lavage fluid consisting of non-degenerative neutrophils [15].

#### Study protocol

Prior to the start of the experimental study, all horses had been maintained on pasture for two months and were clinically healthy. Horses were then stabled for 14 days in a NCE (bedded on wood shavings, fed dusty hay and water ad libitum). On day 15, pulmonary function tests were performed in unsedated horses [9] and entailed measurement of ΔPpl_max_ (average of 15 breaths). Then each horse was sedated, a 2 m Olympus CV 100 bronchoscope (Olympus) was passed nasally into the trachea and 20 mL of a 2% local anesthetic were instilled [9]. Once the airway mucosa was anesthetized, the bronchoscope was advanced and wedged into a distal airway and a bronchoalveolar lavage was performed [9]. BALF samples were maintained on ice until cytological analysis was conducted at the laboratory within 30 minutes of collection. While sedated, each horse was then humanely euthanized by intravenous pentobarbital. The lungs and mediastinal lymph nodes were aseptically removed through a sterile thoracotomy site, rinsed with sterile 0.9% NaCl solution and immediately transported in a sterile container on ice to the laboratory for further processing within a laminar flow hood. Ten mm^3^ samples of mediastinal lymph nodes (containing the cortex and medulla) were snap frozen in an isopentane bath in liquid N_2_ and stored at -80°C for subsequent RNA and protein analysis. Mediastinal lymph node samples that were ten mm^3^ in size and contained the cortex and medulla, were also embedded in OCT compound, snap frozen and stored at -80°C (as described above) until evaluated histologically.

#### Reagents—sample collection

Analgesia and sedation for the BALF collection were provided by intravenous injection (0.1 mg/kg) of xylazine hydrochloride (Tranquived) purchased from Vedco and by intratracheal instillation of 20 mL of 2% lidocaine obtained from Butler. The pentobarbital solution (Fatal Plus, administered at 100 mg/kg IV) was obtained from Vortech. Sterile 0.9% saline, used in the bronchoalveolar lavage and in rinsing of the isolated pulmonary tissues, was obtained from Baxter. The OCT compound for tissue embedding was purchased from VWR Scientific Products.

#### Ethics statement

The horses used in this experiment had been part of a research herd previously established by the PI to study RAO and were known to develop RAO (susceptible horses) or to remain disease free (control horses) when stabled and fed dusty hay (natural challenge environment). All 14 of the horse had been donated to the PI by their owners who understood that the RAO research trials would eventually be terminal. Between experimental trials, all horses were maintained outside on grass pasture (dry lot in winter), provided shelter, fed pelleted hay and concentrate. Fresh water was always available. In this terminal experiment, horses were maintained in a box stall and provided access to free choice dusty and dust-free grass hay, a mineralized salt block and water. Visual contact between horses in the trial was always possible; horses also could view the surrounding farm environment through windows. Health and welfare of the horses was assessed thrice daily by the barn manager with the understanding that should any horse develop respiratory distress, it would be removed from the offending environment immediately and provided veterinary medical assistance administered by the Large Animal Ambulatory Clinicians associated with Cornell University Hospital for Animals. (No horse in the experimental trial developed respiratory distress. No animal in the experimental trial required medical treatment). All experimental procedures were approved by the Institutional Animal Care and Use Committee of Cornell University (Protocol Number: 2009–0006) and were carried out in strict accordance with guidelines established by the National Institutes of Health. All efforts were made to minimize any discomfort of the animals.

### Lymph node histology, immunohistochemistry and cytokine protein quantitation in lymph node homogenates

#### Reagents—lymph node staining

The endogenous biotin-blocking kit and the streptavidin conjugated with Texas Red were purchased from Molecular Probes. Biotinylated goat anti-mouse IgG and VECTASHIELD Mounting Medium with Dapi were obtained from Vector Laboratories. Rabbit anti-CD3 was purchased from Dako. Alexa Fluor 488 goat anti-rabbit IgG was obtained from Life Technologies. Dr. Bettina Wagner (Cornell University) supplied the murine anti-equine monoclonal antibodies (supernatants from clones IL-17A76, 79–3 and 80–1). The monoclonal antibodies were made as part of the US-wide initiative, “The U.S. Veterinary Immune Reagent Network.” The specificity of the equine IL-17A antibodies had been previously tested using various equine recombinant equine cytokines by ELISA. Recombinant cytokines were IgG or IL-4 fusion proteins and included IL-4/IL17, IL-4/IFN-γ, IL-4/IL-5, IL-4/IgG1, IL-2/IgG1 and IL-10/IgG4. The anti-IL-17A antibodies detected the IL-4/IL-17 protein but none of the other IL-4 or IgG fusion proteins. In addition, the IL-17A antibodies were validated in non-stimulated and stimulated equine PBMC by intracellular staining and flow cytometric analysis as previously described [11]. Hybridoma medium to grow the antibodies was purchased from Life Technologies.

#### Staining protocol

Snap-frozen tissue sections (8 μm) of lymph nodes from 5 RAO-affected and 6 control horses were fixed in ice-cold acetone for 10 minutes before endogenous biotin and streptavidin were blocked. After washing in PBS, nonspecific binding was blocked with a mixture of 10% goat serum and 2xcasein for 30 minutes at room temperature. For IL-17 staining, sections were incubated overnight (> 16 hours at 4°C) with a mixture (1:1:1 ratio) of 3 mouse monoclonal antibodies against 3 distinct epitopes of equine IL-17. To assess antibody epitope-specific binding, serum free hybridoma medium was used as an isotype negative control. After washing in PBS, the sections were then incubated with biotinylated goat anti-mouse IgG at 1:200 in PBS for 30 minutes at room temperature followed by incubation with streptavidin conjugated with Texas Red at 1:200 in PBS for 20 minutes at room temperature. For co-labeling of sections with IL-17 and CD3, sections were incubated with the mixture of mouse monoclonal anti-IL-17 and rabbit anti-CD3 at 1:50 overnight (>16 hours at 4°C). Following completion of IL-17 labeling, the sections were further incubated with Alexa Fluor 488 conjugated goat anti-rabbit IgG (1:200 in PBS) for 30 minutes at room temperature. After washing, the stained sections were counterstained and cover-slipped with VECTASHIELD Mounting Medium with DAPI. The fluorescent labeled slides were examined and photographed using Olympus AX 70 compound microscope equipped with MicroFire camera and Picture Frame for image processing and capture (Optronics).

#### Morphologic evaluation

Analysis of hematoxylin- and eosin-stained and CD3 and IL-17 immuno-labeled sections of mediastinal lymph nodes was performed by a pathologist (ELB) who was unaware of the tissue sample source (RAO-affected versus control) of each slide. For each section, ten non-overlapping 400x fields of view were analyzed for the number of eosinophils, neutrophils, pigment-laden macrophages, CD3^+^ immunoreactive cells and for the amount of IL-17 immunofluorescence. Cases were assigned a numeric score between 1 and 4 depending on the intensity or number of specific cells staining for each category. A score of 0 was assigned if no cells were detected in the 10 high-powered fields examined; 1 if 1–3 cells were detected; 2 if 3–10 cells were detected; 3 if multiple clumps or islands of cells were detected and 4, if sheets of cells in multiple high-powered fields were detected.

#### Lymph node homogenates and immunoassays

Previously frozen lymph node samples from the 7 control and 7 RAO-affected horses were transferred to flat bottom Eppendorf vials and weighed. To each sample were added 1 stainless steel 5 mm bead (Qiagen) and 1000 μL of PBS. Each sample was homogenized (Qiagen Tissue Lyser LT) for 5 minutes at an oscillation rate of 40/sec. Samples were centrifuged (ThermoLegend Micro21R) for 3 minutes (14,000 rpm) and supernatants removed and stored on ice until subsequent analysis. A Luminex-based multiplex cytokine assay that had been previously validated [16] was used to quantitate equine IL-4, IL-10 and IL-17. Cytokine concentrations in the supernatants were normalized to 100 mg tissue samples and reported as fluorescence intensity units per mL of homogenate supernatant.

### Microarray and Real-time PCR Assays

#### Reagents—gene expression studies

Kits for isolation of total RNA were purchased from Qiagen. For linear amplification and CY-3 labeling of RNA used in microarrays, the Ambion Amino Allyl Message Amp II kit was obtained from Applied Biosystems. For the RT-PCR assays, DNase I was obtained from Invitrogen, MMLV Reverse Transcriptase was obtained from USB and SYBR Green Mastermix was obtained from Applied Biosystems.

Total RNA was isolated from tissue samples and its quantity and quality were determined using an Agilent Bioanalyzer (Agilent). The RNA integrity numbers (RINs) exceeded 9.5 in 12 samples, equaled 9.2 in one control sample and 8.5 in one RAO-affected sample.

#### Equine Microarray

A custom-designed 8 x 15 equine array developed on the Agilent platform (Agilent Technologies) was used to measure transcripts. As previously reported [17], the equine sequences used in the array were obtained from three National Center for Biotechnology Information databases. Predicted reference sequences (RefSeqs) from the first assembly of the horse whole genome sequence (http://www.broad.mit.edu/mammals/horse) were combined with all horse UNIGENE entries, resulting in 24,628 sequences. All 37,316 available equine-expressed sequence tag sequences were combined with the RefSeq and UNIGENE entries to provide a transcriptome file for the eArray pipeline. Horse-specific mRNA sequences were uploaded into the Agilent eArray custom microarray design pipeline (https://earray.chem.agilent.com/earray/) and duplicate sequences were filtered out. One probe was designed for each input sequence. Each probe received an Agilent quality score of 1 (highest) to 4 (lowest) and a cross-hybridization potential score of 0–1. A total of 14,307 probes with quality/cross-hybridization scores of 1/0, 1/1 or 2/0 were selected for the array [17]. For the array experiment, RNA was linearly amplified, Cy-3 labeled and hybridized to the array slides using standard techniques. Data files were analyzed using GeneSpring GX11 software (Agilent Technologies). Results were quantile normalized and poorly performing probes were filtered out. The P-values for fold-changes were calculated using paired t-tests and the results were exported into a spreadsheet (Microsoft Excel).

#### RT-PCR

Ten genes, differentially expressed in the microarray, were further evaluated by RT-PCR. Following removal of genomic DNA, first strand cDNA was synthesized using M-MLV Reverse Transcriptase following the manufacturer’s protocol. Real time PCR assays using SYBR Green technology (Applied Biosystems) were performed using an Applied Biosystems 7500 Fast Real Time PCR instrument with total reaction volumes of 20μl. Forward and reverse primers (S1 Table) anchored in different exons whenever feasible, were designed. A dissociation curve was performed after each experiment to confirm the amplification of a single product and the absence of contaminating genomic DNA in any of the samples. Standard curves were generated for each gene using known copy numbers of a plasmid containing a gene specific fragment.

### Microarray data mining and statistical analysis

The web-based pathway analysis tool, IPA (Ingenuity Systems Inc., www.ingenuity.com) was used to map gene expression data into relevant biological and molecular pathways. The IPA tool draws upon functional annotations and genome-wide molecular interactions extracted from the scientific literature and stored within the Ingenuity Pathways Knowledge Base (IPKB). The IPKB has been abstracted into a large Global Molecular Network composed of thousands of genes and gene products that interact with each other [18].

For network generation, the IPA tool analyzed genes from the equine microarray data set that were up- or down-regulated ≥ 2-fold (with corrected P-values ≤ 0.05) and for which gene connectivity information existed in the Global Molecular Network. Those focus genes were then sorted in order of gene connectivity relationships to “grow” networks; each network consisted of a maximum of 35 genes. P-values for each network were calculated using the Fisher’s exact test and represented the probability of finding “f” number of focus genes in a set of “n” randomly selected genes from the Global Molecular Network. For the network analysis, molecule functions with a corrected P ≤ 0.05 (maximum false discovery rate of 5%) were considered in the final analysis [18].

Differences between the two treatment groups in pulmonary function tests, in histological scores of lymph node tissue and in immunoassay results of lymph node homogenates were analyzed using the Student’s t-test with significance set a P ≤ 0.05. Commercial software was used for statistical analyses (Statistix, version 8.0, Analytical Software, Tallahassee, FL).

## Results

### Animal studies

Following NCE, all 7 RAO-susceptible horses developed airway obstruction (mean ± SD, ΔPpl_max_ = 42 ± 10 cm H_2_O, P < 0.002) and airway neutrophilia (mean ± SD, BALF neutrophil percentages = 47 ± 15, P < 0.002). Control horses failed to develop signs of RAO (Δ Ppl_max_ = 6.5 ± 3 cm H_2_O; BALF neutrophil percentages = 6 ± 3).

### Differentially expressed genes in microarray

Compared to controls, 219 genes were differentially expressed ≥ 2-fold (P < 0.05) in the mediastinal lymph nodes of RAO-affected horses: 165 genes were up-regulated, 54 genes were down-regulated (S2 Table). Biological processes and/or molecular functions were assigned to 189 of the 219 genes. Listed in Table 1 are the 15 top up-regulated genes, nine of which are associated with inflammatory, acute phase and/or chemotactic responses (DEFB4A, CD163, SAA1, SAA2, PRG4, TNFAIP6, IL8, IL22 and CXCL17). The remaining up-regulated genes are involved in signal transduction including G-protein coupled receptor signaling (EFCAB10 and DEFB4A), ciliary function (EFCAB10, SNTN), oxido-reductive processes (GSTA1) and tissue remodeling (MMP1). Of the top 15 down-regulated genes (Table 2), 5 are involved in immune responses (VPREB1, PRG3, PRG2, KIR3DL, IL4), 3 in oxidative stress responses (TP53i11, CYP2C92, RGS13), 2 with cellular calcium ion homeostasis (CALCB, CSRP3), 2 with cell cycle/division and proliferation (CDK20, TP53i11) and 1 with apoptosis and endothelin maturation (ECE1). Of special note was the finding that IL22 expression was up-regulated 5-fold and that IL4 expression was down-regulated 3-fold in the lymph nodes of diseased horses compared to controls.

**Table 1:** Top 15 up-regulated molecules in mediastinal lymph nodes from RAO-affected horses.

Accession No.	Gene		Fold-change	Biological or Molecular Processes
NR_027068	EFCAB10	EF-hand calcium binding domain 10	10.8↑	Signal transduction; regulation of phosphorylation
NM_004942	DEFB4A	Beta defensin 3	10.5 ↑	Immune response; chemotaxis; G-protein coupled receptor signaling
NM_145750	GSTA1	Glutathione s-transferase alpha 1	8.5 ↑	Glutathione metabolic process; oxido-reduction pathways
NM_004244	CD163	Hemoglobin/Haptoglobin scavenge receptor	7.5 ↑	Inflammatory and acute phase responses
NM_199161	SAA1	Serum amyloid A1	7.4 ↑	Inflammatory (negative regulation) and acute phase responses
NM_001080537	SNTN	Sentan, cilia apical structure protein	6.8 ↑	Ciliary function, respiratory epithelium
NM_005807	PRG4	Proteoglycan 4	6.4 ↑	Immune response; cell proliferation
NM_002421	MMP1	Matrix metallopeptidases 1 (collagenase)	6.2 ↑	Proteolysis; collagen catabolic process
NM_007115	TNFAIP6	Tumor necrosis factor α-induced protein 6	5.4 ↑	Inflammatory response; signal transduction
NM_003018	SFTPC	Surfactant protein C	5.2 ↑	Pulmonary gas exchange
NM_000584	IL8	Interleukin-8	5.1 ↑	Inflammatory and immune responses; chemotaxis
NM_017697	ESRP1	Epithelial splicing regulatory protein 1	5.1 ↑	mRNA processing
NM_020525	IL22	Interleukin-22	5.0 ↑	Inflammatory and acute phase responses
NM_030754	SAA2	Serum amyloid A2	4.8 ↑	Chemotaxis; acute phase response
NM_198477	CXCL17	Chemokine (C-X-C motif) ligand 17	4.8 ↑	Immune response; chemotaxis

**Table 2:** Top 15 down-regulated molecules in mediastinal lymph nodes from RAO-affected horses.

Accession No.	Gene		Fold-change	Biological or Molecular Processes
NM_007128	VPREB1	Pre-B lymphocyte 1	12.1↓	Immune response (Ig gene rearrangements B cell)
NM_178432	CDK20	Cyclin dependent kinase 20	4.0 ↓	Cell cycle, division; phosphorylation
NM_001080434	LMTK3	Lemur tyrosine kinase 3	4.0 ↓	Negative regulation of phosphatase activity
NM_006093	PRG3	Proteoglycan 3	3.8 ↓	Immune response; regulation of IL-8, histamine, leukotriene biosynthesis
NM_002728	PRG2	Proteoglycan 2	3.8 ↓	Immune response; defense response to bacteria
NM_013289	KIR3DL	Killer cell immunoglobulin-like receptor	3.4 ↓	Immune response
NM_000728	CALCB	Calcitonin-related polypeptide beta	3.3 ↓	Signal transduction; cellular calcium homeostasis
NM_001082519	IL4	Interleukin-4	3.1 ↓	Immune response
NM_006034	TP53i11	Tumor protein p53 inducible protein 11	3.1 ↓	Negative regulation of cell proliferation; response to stress
NM_021813	BACH2	Basic leucine zipper transcription factor 2	3.0 ↓	Regulation of transcription
NM_001397	ECE1	Endothelin converting enzyme 1	3.0 ↓	Apoptosis; endothelin maturation
NM_000772	CYP2C92	Cytochrome P_450_ family 2 subfamily C polypeptide 9	2.9 ↓	Cellular amide metabolism; oxidation-reduction process
NM_031476	CSRP3	Cysteine and glycine-rich protein 3	2.8 ↓	Cell differentiation; calcium ion homeostasis; blood vessel remodeling
NM_006121	RGS13	Regulator of G-protein signaling 13	2.7 ↓	Complement activation; response to oxidative stress
NM_020914↓	RNF213	Ring finger protein 213	2.7 ↓	Protein ubiquitination

#### Validation of microarray by RT-PCR assays

The fold-change in expression of ten genes assayed by real time PCR and microarray were compared (S3 Table). The direction of change in gene expression data obtained with the RT-PCR data was consistent with that obtained in the microarray assays.

#### Network and canonical pathways

In analyzing the data *in toto*, inflammation was the top disease process identified with significant expression of 61 molecules (P-values ranged from 3.5x10^-12^ to 4.7 x 10^−3^). In considering the top molecular and cellular functions represented, 49 of the molecules in the microarray expression data were associated with cell-to-cell signaling and interactions and/or immune cell trafficking (P-values ranged from 1.9 x 10^−9^ to 4.9 x10^-3^).

**Fig 1:**
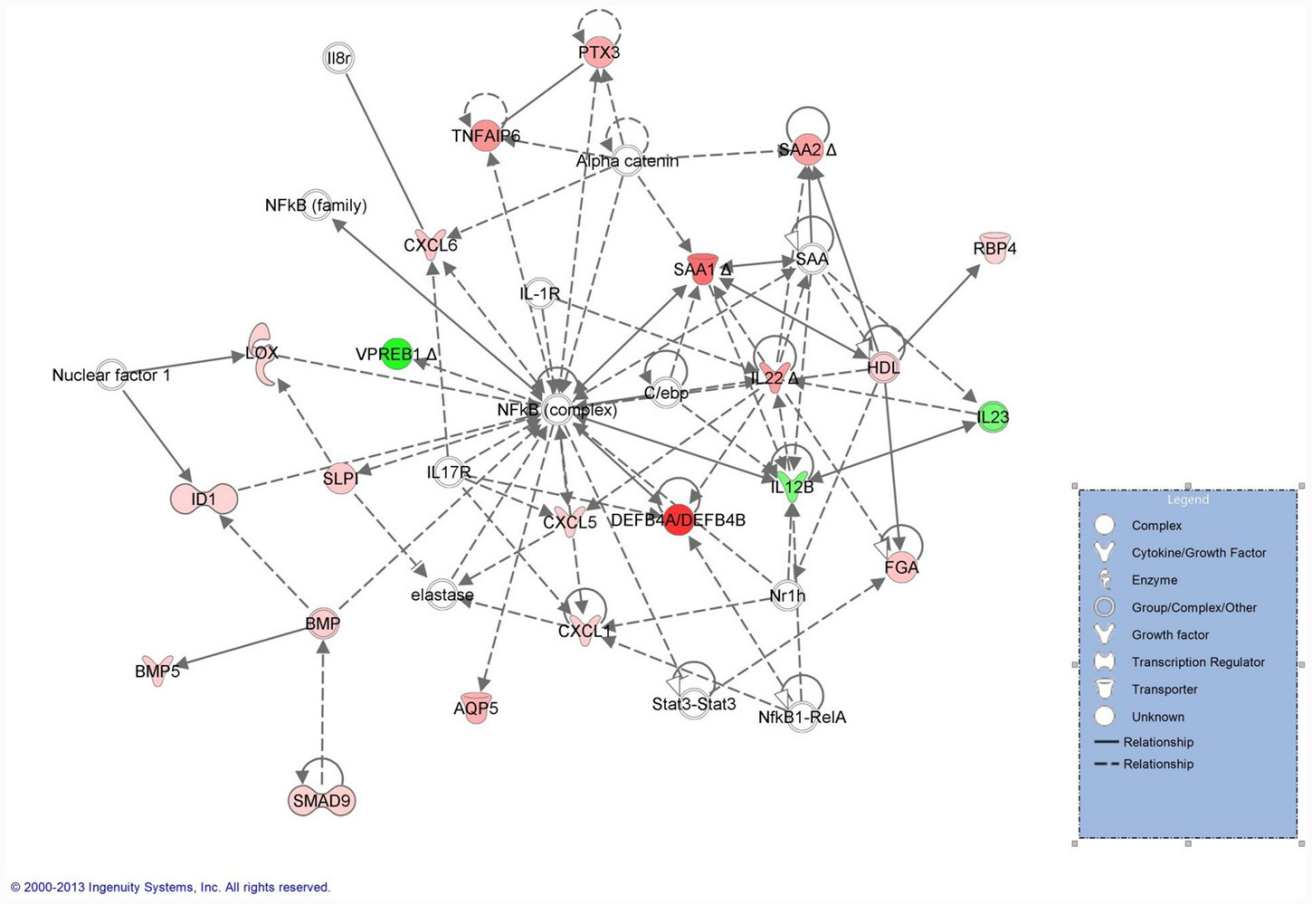
Global gene expression profile in mediastinal lymph nodes from RAO-affected horses supports an inflammatory network focused on NF-κB. The relationship between 19 of the highly expressed (focus molecules) from the microarray data set and other molecules, as derived from the Ingenuity Pathways Knowledge Base, is shown. Up-regulated genes are shown in red and down-regulated genes are in green. For molecules depicted in white, expression data were either not measured or were not significantly changed in the equine microarray data. (The networks were generated through the use of QIAGEN’s Ingenuity Pathway Analysis (IPA QIAGEN Redwood City, www.qiagen.com/ingenuity).

The network shown in Fig 1 depicts the relationship between 19 of the highly expressed (focus) molecules from the microarray data set and their interactions with 6 other molecules derived from the Global Molecular Network of IPKB. At the center of the network is the NF-κB complex. Molecules shown in red are up-regulated; those in green are down-regulated with the color intensity reflecting the magnitude of change in gene expression. For genes shown in white, microarray expression data were lacking either because of inadequate probe design (c/ebp) or because the magnitude of gene expression failed to meet network inclusion criteria (≥ 2-fold change or corrected P-value ≤ 0.05). This occurred with Catenin alpha 1 (CTNNA1; 1.5-fold ↑; P = 0.003); Interleukin-1 receptor 2 (IL1R2; 2.4-fold ↑; P = 0.06); Interleukin-1 receptor 1(IL1R1; 1.2-fold ↓; P = 0.53); Nuclear factor 1 (1.6-fold ↑; P = 0.072); Interleukin-17 receptor A (IL17RA; 1.1-fold ↓; P = 0.96); Interleukin-8 receptor (IL8R; 1.1-fold ↓; P = 0.27); Signal transducer and activator of transcription (STAT3; 1.5-fold ↑; P = 0.48); Nuclear receptor 1h (Nr1h; 1.3-fold ↓; P = 0.62); v-rel reticuloendotheliosis viral oncogene homolog (RELA, p65; 1.4-fold ↑; P = 0.09) and elastase (Metallomatrix protease 12, 1.5-fold ↑; P = 0.42).

**Fig 2:**
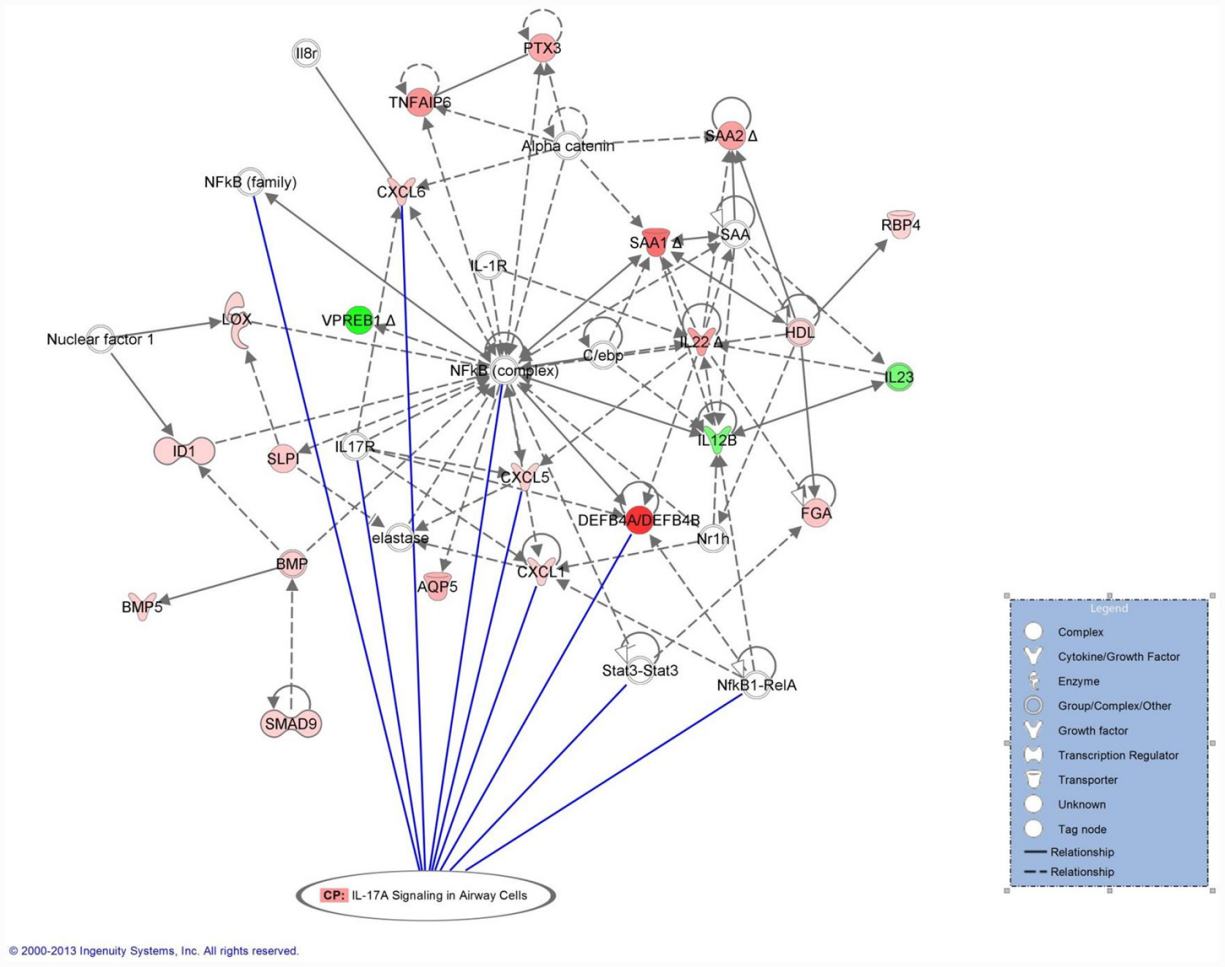
Relationship of the canonical IL-17 pathway to the NF-κB-based network. Following activation of its receptor, IL-17 promotes transcription of many pro-inflammatory genes including CXCL1, CXCL5, CXCL6 and beta-defensin, indicated by the solid lines. (The networks were generated through the use of QIAGEN’s Ingenuity Pathway Analysis (IPA QIAGEN Redwood City, www.qiagen.com/ingenuity).

The relationship of the network to canonical pathways was evaluated and demonstrated a significant association (P = 1.7 x 10^−7^) with the IL-17 signaling pathway (Fig 2) although neither real time PCR analysis nor microarray assay demonstrated a significant up-regulation of IL-17 in mediastinal lymph node samples of RAO-affected horses.

### Histology of mediastinal lymph node sections

**Fig 3:**
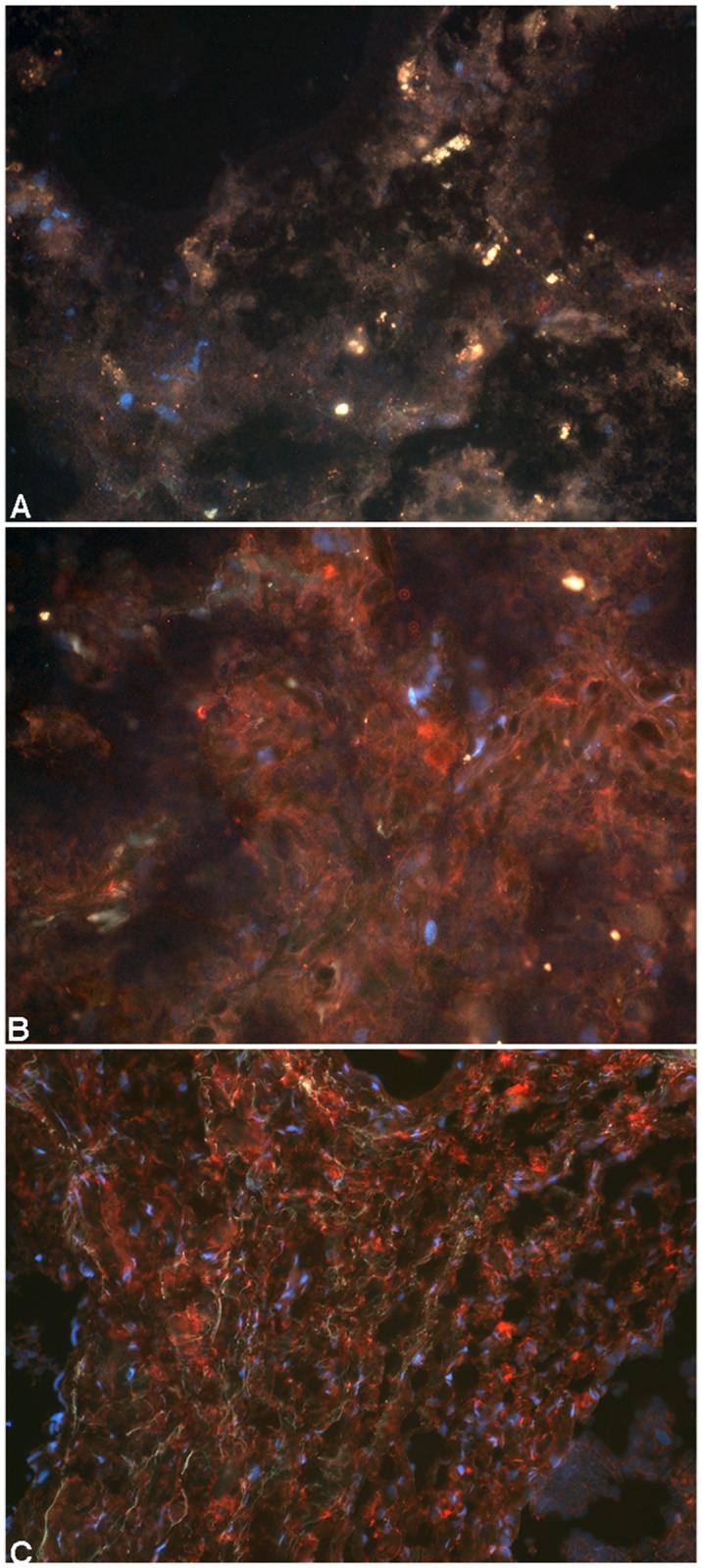
Enhanced IL-17 immunoreactivity in lymph node sections of RAO-affected horses. (A) Lymph node tissue from a control horse shows no evidence of IL-17 immunofluorescence. (B) Lymph node tissue from one RAO-affected horse exhibits widespread extracellular IL-17 immunofluorescence (red staining). (C) Lymph node tissue from another RAO-affected horse exhibits denser, widespread IL-17 immunofluorescence (red). All photomicrographs, 200X, are counterstained with Texas Red and Dapi.

The mean (± SD) eosinophil score for the 6 control horses was 2.0 (± 0.9) and was significantly greater than that of 5 RAO-affected horses (mean = 0.4 ± 0.5; P = 0.007). The average number of pigment-laden macrophages in control horses (1.3 ± 0.8) did not differ from that of the diseased horses (mean = 1.0 ± 0.7). Neutrophils were not prominent in any of the sections but when they occurred, were found only in the RAO-affected horses (mean score = 0.8 ± 0.8; P > 0.05). IL-17 staining was not present in lymph node sections from 3 control horses (scores = 0); in the remaining 3 controls, IL-17 staining intensity was faint (scores = 1). Lymph node sections from the 5 RAO-affected horses had evidence of IL-17 staining (scores = 1, 2, 3, 4, 4). As demonstrated in Fig 3, the intensity of IL-17 staining was significantly greater in tissue sections from the RAO-affected horses (mean score = 2.8 ±1.3; P = 0.01) compared to intensity of staining in the controls (mean score = 0.5 ± 1.1). Overall, the majority of the IL-17 staining was located in the cortical regions of the lymph nodes with lesser staining found in the medullary regions. All horses had a large number of CD3^+^ immunoreactive cells in the lymph node sections, but because of artifact with the frozen tissue processing, neither an adequate comparison between the two groups nor localization of IL-17 to CD3^+^ cells was possible. Because of this difficulty, co-localization of IL-17 to other cells types (e.g. macrophages, neutrophils, B-cells) was not attempted.

### Cytokine protein expression in lymph node homogenates

Interleukin-17 was detected in all 14 samples. The mean (± SD) fluorescence intensity of the supernatants from the 7 control horses, 2.4 (±0.9) U/mL, was less than the mean fluorescence intensity in the supernatants of the 7 RAO-affected horses of 9.5 (±13.5) U/mL (P = 0.1). Neither IL-4 nor IL-10 was detected in any of the 14 supernatant samples from the lymph node homogenates.

## Discussion

We are unaware of any other studies in which the global gene expression profiles of mediastinal lymph nodes from RAO-affected horses have been examined. In undertaking this investigation, we sought to gain insight into the immunological basis underlying chronic, active RAO in horses. We evaluated transcriptomes of mediastinal lymph node cells as a reflection of the immunological events occurring within the lung. We detected differential expression of at least 219 genes, 189 of which are functionally annotated. Using a network-based bioinformatics approach, we found that many of the differentially regulated genes were associated with inflammatory responses and involved cell-to-cell trafficking and communication. Transcriptome expression, which included an up-regulation of IL-22 and selected CXC-chemokines, coupled with a down-regulation of IL-4, supported the involvement of the IL-17 pathway even though IL-17 mRNA expression itself was not increased in lymph nodes of diseased horses relative to controls. As concurrent increases in IL-17 gene and protein expression do not always occur physiologically [19], we assessed IL-17 protein immunoreactivity in lymph node sections and in supernatants from lymph node homogenates. We found enhanced IL-17 protein staining intensity in tissues and IL-17 protein concentrations in supernatant homogenates from all diseased horses. We were unable to co-localize IL-17 to CD3^+^ cells. Collectively our data suggest that the chronic inflammatory response in horses with RAO involves IL-17 and not IL-4.

### Global gene expression studies in RAO-affected horses

Previous investigators have examined global gene expression profiles of selected tissues of RAO-affected horses. Ramery et al. (2008), using a human microarray (Affymetrix U133 Plus 2.0 GeneChip), assessed transcriptomes of BALF cells obtained from RAO-susceptible horses maintained in a natural challenge environment for at least 7 days. They reported [20] that 3 genes were up-regulated ≥ 4.0-fold in the BALF cells of diseased horses: Tribbles homolog 1 (*Drosophila*,TRIB1); Growth arrest and DNA damage-inducible alpha (GADD45A) and Pentraxin-related gene rapidly induced by IL-1β (PTX3). In our study, these 3 genes were also expressed in the mediastinal lymph nodes but only PTX3 was significantly up-regulated in tissues from the diseased horses (4.3-fold, P = 0.047). Pentraxin, an acute-phase protein, is synthesized by equine macrophages, neutrophils and bronchial epithelial cells [21] in response to infectious and inflammatory stimuli [22]. As a soluble pattern recognition molecule of the innate immune system, PTX3 modulates the severity of the inflammatory reaction and seemingly exerts a “protective” role in murine lung injury models [22]. In RAO-affected horses, it is likely that PTX3 gene expression was up-regulated following exposure to LPS or β-glucans contained within the inhaled dusty hay particulates [23,24]. Ramery (2008) also reported that 10 genes were down-regulated ≥ 4-fold in the BALF cells of RAO-affected horses: RAB6A member RAS oncogene family (RAB6A); Eukaryotic translation initiation factor 5A (EIF5A); Myristoylated alanine-rich protein kinase C substrate (MARCKS); CUG triplet repeat, RNA binding protein 2 (CUGBP2); Zinc finger protein 148 (ZNF148); Signal transducer and activator of transcription 3 (STAT3); SAR1 gene homolog A (*S*. *cerevisiae*; SAR1A); IQ motif containing GTPase activating protein 1 (IQGAP1); RAP2A, member of RAS oncogene family (RAP2A); RAP2B member of RAS oncogene family (RAP2B) and Membrane associated DNA binding protein (MNAB also known as RC3H2). In the present study we failed to detect a significant difference in the expression of these same genes between diseased and control horses, a finding that may reflect the difference in the cell populations evaluated, e.g. BALF cells versus lymph node cells.

Using suppression subtractive hybridization techniques, Lavoie et al. (2012) identified 147 differentially expressed genes in equine peripheral lung biopsies obtained from RAO-affected horses. Although these investigators [25] focused primarily on genes associated with smooth muscle biology and remodeling (14 were differentially expressed), they noted that several genes associated with inflammation were overexpressed in the lungs of diseased horses. These included LTA4H (leukotriene A4 hydrolase, an enzyme which metabolizes LTA4 into LTB4); PGFS (Prostaglandin F synthase, an enzyme that reduces PGD2 and PGH2 to PGF2); PTGDR (Prostaglandin D2 receptor), and CNOT7 (CCR4-NOT transcription complex, a repressor of the retinoid X beta receptor, Rxrb, that forms a heterodimeric complex with the nuclear receptors PPARs—peroxisome proliferator-activated receptor). In the current study, LTA4H and PTGDR were not assayed and the fold-changes in expression of PFGS and CNOT7 were not statistically significant. In comparing our study with that of Lavoie and colleagues (2012), we noted that none of our top 15 up- or down-regulated genes were differentially expressed in their study (S2 Table). This was especially surprising for IL-8, a chemokine which was up-regulated 5-fold in the current study and has been previously demonstrated to be up-regulated in the bronchial epithelial and BALF cells of RAO-affected horses [6,9,10,26,27,28].

### Network analysis—NF-κB and IL-17

The pattern of differential gene expression was consistent with an inflammatory disorder centered about NF-κB (Fig 1), a transcription factor that exerts a pivotal role in the regulation of genes involved in the innate and adaptive immune responses, cellular proliferation and apoptosis [29]. Depending upon the specific tissue examined, the NF-κB complex is a hetero- or homodimer consisting of subunits p50, p52, p65/RelA, RelB or c-Rel/Rel. The p65 and p50 subunits are expressed in a variety of cells but c-Rel gene expression is confined to hematopoietic cells and lymphoid tissue [30]. The protypical NF-κB complex consists of the p50/p65 heterodimer except in the bronchial epithelium and BALF cells of RAO-affected horses where the NF-κB complex is comprised predominantly of p65 homodimers [31,32]. Although we failed to detect significant increases in p65 (RelA) or in c-Rel gene expression in our study, we also did not perform functional assays that would have allowed us to quantitate activation of specific subunits of NF-κB.

Network analysis suggested that the inflammatory process was driven, in part, by IL-17 (Fig 2). This cytokine, originally thought to be produced exclusively by a subset of T-helper cells, is now known to be secreted by a variety of cells. In humans, cellular sources of IL-17 include CD4^+^, CD8^+^, γδT cells, NK cells, NKT cells, lymphoid tissue-inducer (LTi) and LTi-like cells as well as granulocytes, macrophages and dendritic cells [33]. Interleukin-17 signals through a receptor complex (IL-17RA/IL17RC) which is expressed on a variety of cells including leukocytes, epithelial cells, endothelial cells, fibroblasts and astrocytes [34]. Receptor activation induces transcription of CXC-chemokines such as CXCL1, CXCL2, CXCL5, CXCL6 and CXCL8 (which promote neutrophil influx) and other pro-inflammatory cytokines like IL-6, IL-1β and TNF-α. Interleukin-6 transcription further amplifies the inflammatory response by activating the acute phase genes [35]. IL-17, alone or in combination with TNF-α, also stabilizes the mRNA of transcribed chemokines, prolonging the inflammatory response [36]. Additional target genes of IL-17 (CXCL9, CXCL10 and CCL20) exert chemotactic activity for lymphocytes, dendritic cells and other immune cells that contribute to the inflammatory response [35].

One of the limitations of our network analysis can be a failure to realize the extent to which gene products internal or external to the proposed network interact with the IL-17 pathway and contribute to the inflammatory process. One such example is IL-22, a member of the IL-10 cytokine family that plays a critical role in inflammation, immune surveillance and tissue homeostasis at mucosal sites [37]. Although IL-22 is co-produced by Th17 cells, IL-22 is also generated by Th22 cells, γδT cells, innate lymphoid cells and alveolar macrophages independently of IL-17 [38,39]. Interleukin-22 activates target cells through a heterodimeric receptor complex comprised of IL-22R1 and IL-10R2 and signals intracellularly through several pathways including STAT1, 3 and 5 [40]. Similar to IL-17, IL-22 also activates the JAK/STAT, ERK, JNK and p38 MAPK pathways with subsequent gene expression [40]. Interestingly, IL-22 exerts pro- or anti-inflammatory functions depending upon the existing cellular and cytokine milieu: In the absence of IL-17, IL-22 protects against lung pathology by increasing airway epithelial cell proliferation, promoting the integrity of the epithelial barrier [37]. In the presence of IL-17, the pro-inflammatory properties of IL-22 are manifested and include synergistic expression of inflammatory cytokines, chemokines and neutrophil recruitment as well as suppression of the anti-apoptotic effects of IL-22 on epithelial cells [37,38]. In the current study, the up-regulation of IL-22 gene expression in the presence of enhanced protein expression of IL-17 suggests that IL-22 was exerting a pro-inflammatory role in RAO.

In contrast, an example of a gene product not shown in the network analysis that likely impacted on the IL-17 pathway and the inflammatory process of RAO is IL-4. Recently Choy and colleagues (2015) reported on their studies of gene expression profiles in endobronchial samples from human asthmatics and from lung samples in a murine model of allergen-induced asthma. They found that gene signature profiles of Th2 (IL-13, IL-4) and Th17 (CXCL1, CXCL2, CXCL3, IL-8, CSF3) immune responses were inversely correlated in these samples suggesting a reciprocal regulation of these two pathways in asthma [41]. In the current study, there was a significant down regulation of IL-4 gene expression concurrent with a lack of detectable IL-4 protein in lymph node samples of the diseased horses. This finding suggests the existence of a similar reciprocal regulation of the Th2 and Th17 pathways in chronic RAO.

IL-17 producing cells are typically positioned at mucosal interfaces where they exert a critical function in the killing of extracellular bacteria. The cytokine is also linked to the pathogenesis of autoimmune disorders—rheumatoid arthritis, psoriasis, and systemic lupus erythematosus [35]—as well as inflammatory disorders such as neutrophilic asthma and chronic obstructive pulmonary disease [42,43]. In healthy humans, pulmonary instillation of endotoxin results in a significant increase in the number of IL-17 producing CD4^+^ cells in the airways as well as an increase in the IL-17 protein concentrations in the BALF [44]. Agricultural workers involved in cattle and swine farming operations exhibit a higher percentage of T-cells expressing IL-17 and IFN-γ in the peripheral blood suggesting that agricultural dust exposure causes a polarization of the T-cell responses towards a Th-17 response [45]. In a murine model of agricultural (occupational) asthma, repetitive nasal instillation of organic dust extract derived from animal farming operations increased IL-17, IL-1β and IL-6 protein concentrations in lung homogenates as well as induced a neutrophilic infiltrate in the lungs [45].

Although IL-17 involvement in certain pulmonary inflammatory responses has been established, few studies have examined the concurrent cytological or gene-expression responses in the draining (mediastinal) lymph nodes during these conditions. In a murine model of chronic asthma induced by ovalbumin exposure, Lee (2013) reported a significant increase in the percentage of Th17 cells in both the lymph node and lungs of affected mice. Interestingly, this cellular response exceeded the percentage change in Th1 or Th2 cells in those same tissues [46]. Unfortunately, global gene expression profiles of the mediastinal lymph nodes were not reported making it difficult to discern whether the augmented Th17 response in the lymph node also contributed to an inflammatory gene expression profile in the lymph nodes. In a murine model of sterile inflammation induced by photodynamic treatment of induced-tumors, increased intracellular IL-17 staining and increased numbers of Th17 cells were documented in the draining axillary and brachial lymph nodes of tumor-bearing mice. Interestingly, lymph nodes from the affected mice displayed a neutrophilic infiltrate which the investigators attributed to an interaction of the CCR2 on the neutrophil with its ligand, CXCL2, the latter being induced by IL-17 and IL-1β expression [47]. In the current study (an equine model of “sterile pulmonary inflammation”) we did not observe a significant neutrophilic infiltrate in the mediastinal lymph nodes of diseased horses despite strong IL-17 staining intensity. Although this finding requires further investigation, one plausible explanation is that the circulating neutrophils failed to express CCR2 which would have allowed them to interact with the up-regulated CXCL2 and infiltrate into the lymph node tissue. This was supported by our finding that the expression of CCR2 was not significantly altered in the RAO-affected horses (1.1-fold change, P = 0.551). Additional studies are warranted to ascertain the cellular source of IL-17 in the pulmonary lymphoid tissue of horses with RAO.

In conclusion, based upon analysis of global gene expression profiles, immunohistochemical staining of mediastinal lymph nodes and immunoassay of lymph node homogenates isolated from horses with chronic RAO, we provide data suggesting that one component of the chronic inflammatory response of RAO involves IL-17. Understanding the temporal involvement of IL-17 in the progression of this disease may encourage the development of new therapeutic approaches for management of RAO in horses and occupational asthma in humans.

## Supporting Information

S1 Table Sequences of primer pairs used in RT-PCR analysis. (DOCX)

S2 Table Identification and functional classification of differentially expressed genes in mediastinal lymph node tissues of horses with RAO as compared to control horses. (DOCX)

S3 Table Fold-change (RAO:control) in expression of 10 genes measured by RT-PCR and microarray assays. (DOCX)
