# Supplementary material for: Differential Gene Expression Profiles and Selected Cytokine Protein Analysis of Mediastinal Lymph Nodes of Horses with Chronic Recurrent Airway Obstruction (RAO) Support an Interleukin-17 Immune Response
Source: PLoS One. 2015 Nov 12;10(11):e0142622. doi: 10.1371/journal.pone.0142622 (PMC4642978; doi:10.1371/journal.pone.0142622)
Supplement: S1 Table — (DOCX) [file pone.0142622.s001.docx]

**Table S1. Sequences of primer pairs used in RT-PCR analysis**

| **Primary Accession No.** | **Gene** | | **Forward 5’ → 3’** | **Reverse 5’→ 3’** |
| --- | --- | --- | --- | --- |
| NM_001083951 | IL8 | Interleukin-8 | GCTTTCTGCAGCTCTGTGTG | AAGGTTTGGAGTGCGTCTTG |
| ENSECAT00000007775 | DEFB4A | Beta defensin 3 | TCTCCTTGTGTTCCTCGTTG | TGACCCTGGAAGGCACTTAG |
| NM_001081928 | CALCB | Calcitonin-related polypeptide beta | TGAAGGCATATGTGCAGAGG | ATCTCTTCTGGGCAGTGAGG |
| ENSECAT00000020379 | CD163 | Hemoglobin scavenge receptor | TCAGTGCCTGTTTGATCACC | TCCTGGACTTTCACCTCCAC |
| AY246746 | PRG4 | Proteoglycan 4 | CACACACCATCCGAATTCAC | AACCGTATCGGGAAGTCCTC |
| ENSECAT00000024801 | TP53i11 | Tumor protein p53 inducible protein 11 | CAGTTCTTGGTGGTCACTGC | TTCGGCCGACTTGGTAATAG |
| AF053497 | CXCL1 | Chemokine (C-X-C motif) ligand 1 | GAACATCCAGAGCGTGAAGG | GGGCTTCAGGATTGAGACAAG |
| NM_001081886 | CXCL6 | Chemokine (C-X-C motif) ligand 6 | AGAGAACTGCGTTGCATGTG | GGGTCCAGACAGACTTCCTTC |
| ENSECAT00000019533 | IL17A | Interleukin-17 | CAGATCCAGCCCTTCTTCAG | ACCCACAGCGGCATATAGTC |
| ENSECAT00000014744 | RELA | NFκB subunit p65 | GTCGCCTGTCCTTTCTCATC | TGTCCTCTTTCTGCACCTTG |
