# Supplementary material for: Differential Gene Expression Profiles and Selected Cytokine Protein Analysis of Mediastinal Lymph Nodes of Horses with Chronic Recurrent Airway Obstruction (RAO) Support an Interleukin-17 Immune Response
Source: PLoS One. 2015 Nov 12;10(11):e0142622. doi: 10.1371/journal.pone.0142622 (PMC4642978; doi:10.1371/journal.pone.0142622)
Supplement: S2 Table — (DOCX) [file pone.0142622.s002.docx]

**Table S2. Identification and functional classification of differentially expressed genes in mediastinal lymph node tissues of horses with RAO as compared to control horses.**

| Human Ref Seq | Fold -change | Name of Gene | Biological Process | Molecular Functions |
| --- | --- | --- | --- | --- |
| NM_000014 | 2.64 | Alpha-2 macroglobulin | Acute phase response | Protein binding: IL-8, IL-1, TNF-α,TGF-β |
| NM_021804 | 2.46 | Angiotensin I converting enzyme | Blood pressure regulation, ROS metabolism, cytokine production, inflammatory response | Peptidase activity |
| NM_202000 | 4.51 | Acyl-CoA synthetase medium-chain family member | Fatty acid metabolic process | ATP binding, fatty acid ligase activity |
| NM_006988 | 2.20 | ADAM metallopeptidase with thrombospondin type 1 motif 1 | Integrin-mediated signaling pathway; negative regulator of cell proliferation | Peptidase activity |
| NM_024758 | 2.47 | Agmatine ureohydrolase | Cellular nitrogen compound metabolism | Hydrolase activity (C-N bonds) |
| NM_006408 | 3.29 | Anterior gradient homolog 2 | Mucus secretion | Protein binding |
| NM_176813 | 3.67 | Anterior gradient homolog 3 |  | alpha dystroglycan binding |
| NM_001620 | 2.50 | AHNAK nucleoprotein | Nervous system development | Protein binding |
| NM_020661 | -2.08 | Activation-induced cytidine deaminase | B-cell differentiation (isotype switching) | Hydrolase activity (C-N bonds) |
| NM_013410 | 2.09 | Adenylate kinase 4 | Development of brain, liver; nucleotide phosphorylation | ATP binding; adenylate kinase activity |
| NM_152327 | 2.76 | Adenylate kinase 7 | Nucleobase-containing compound metabolism | ATP binding; adenylate kinase activity |
| NM_000689 | 2.02 | Aldehyde dehydrogenase 1 family, member L2 | Oxidation-reduction process; metabolism | Oxidoreductase activity |
| NM_198098 | 3.49 | Aquaporin 1 | Cell response to stress, hypoxia; negative regulator of apoptosis | Water, potassium ion transmembrane transporter |
| NM_001651 | 4.05 | Aquaporin 5 | Transporter water, CO_2_ | Water channel activity, protein binding |
| NM_021226 | -2.17 | Rho GTPase activating protein | Small GTPase-mediated signal transduction | GTPase activator activity; phospholipid binding |
| NM_017680 | 4.24 | Asporin | Negative regulator of TGF-β receptor signaling pathway | Protein binding; calcium binding |
| NM_000705 | -2.01 | ATPase, H^+^/K^+^ exchanging beta polypeptide | Ion transport; ATP catabolic process | H^+/^K^+^ exchange |
| NM_021813 | -2.98 | BTB and CNC homology 1, basic leucine zipper transcription factor 2 | Regulation of transcription | DNA binding; protein binding |
| NM_000055 | 3.91 | Butyrlcholinesterase | Metabolism of choline; negative regulation of cell proliferation | Acetylcholinesterase activty; beta-amyloid binding |
| NM_021073 | 2.50 | Bone morphogenetic protein 5 | Cartilage development; male genitalia development | BMP receptor binding; cytokine activity; growth factor activity |
| NM_005182 | -2.33 | Carbonic anhydrase 7 | Metabolic process - one carbon | Carbonate dehydratase activity |
| NM_000728 | -3.28 | Calcitonin-related polypeptide beta | Signal transduction; cell Ca^+^ homeostasis | Neuropeptide activity |
| NM_000070 | 2.44 | Calpain 3 (p94) | Cell response to Ca^+2^; positive regulation of NF-kB transcription factor activity; regulation of I-kappa B kinase/NF-kappa cascade | Calcium ion binding; protein binding; signal transducer activity |
| NM_001753 | 2.42 | Caveolin 1 | Negative regulation of MAPK cascade; Ca^+2^ homeostasis, transport; | Kinase binding; protein binding |
| NM_178432 | -4.05 | Cyclin-dependent kinase 20 | Cell cycle; protein phosphorylation | ATP binding; kinase activity; transferase activity |
| NM_000591 | 2.24 | CD 14 molecule | Inflammatory, innate immune responses; response to LPS, | LPS binding; lipoteichoic acid binding; peptidoglycan receptor activity |
| NM_004244 | 7.49 | CD 163 molecule | Acute phase, inflammatory response | Protein binding; receptor activity |
| NM_001763 | -2.20 | CD1a molecule | Immune response: antigen processing and presentation | Protein binding |
| NM_001764 | -2.10 | CD1b molecule | Immune response; antigen processing and presentation | Protein binding |
| NM_030893 | -2.22 | CD1e molecule | Immune response; antigen processing and  presentation |  |
| NM_001773 | 2.02 | CD34 molecule | Immune response: positive regulation of IL-10; TGF-β production; negative regulation of TNF-α gene expression, | Transcription factor binding |
| NM_020549 | -2.26 | Choline-O-acetyltransferase | Acetylcholine synthesis; response to hypoxia | Choline binding; transferase activity (acyl) |
| NM_014358 | 2.18 | C-type lectin domain family 4, member E | Immune response: positive regulation of cytokine secretion | Protein binding; receptor binding |
| NM_014461 | 2.18 | Contactin 6 | Cell adhesion; CNS development; Notch  signaling pathway |  |
| NM_001845 | 2.91 | Collagen type IV, alpha 1 | Epithelial Cell differentiation; angiogenesis | Protein binding; extracellular matrix constituent |
| NM_020351 | 3.72 | Collagen type VIII, alpha 1 | Angiogenesis; cell adhesion; epithelial cell proliferation |  |
| NM_001855 | 2.08 | Collagen type XV, alpha 1 | Angiogenesis; cell adhesion; signal transduction | Binding; extracellular matrix structural constituent |
| NM_001037763 | 2.82 | Collagen type XXVIII, alpha 1 | Cell adhesion; neg reg endopeptidase activity | Peptidase inhibitor activity |
| NM_020361 | 2.71 | Carboxypeptidase A6 | Proteolysis | Carboxypeptidase activity; hydrolase activity |
| NM_001873 | 2.89 | Carboxypeptidase E | Protein modification, proteolysis | Protein binding; peptidase activity |
| NM_003296 | 2.41 | cysteine-rich secretory protein 3 | Immune response-innate |  |
| NM_031476 | 2.54 | cystein-rich secretory protein LCCL domain containing 2 | Extracellular matrix organization | Glycosaminoglycan binding |
| NM_000567 | 2.46 | C-reactive protein, pentraxin-related | Acute phase, inflammatory response | Protein binding; lipoprotein binding |
| NM_003476 | -2.83 | Cysteine and glycine-rich protein 3 | Blood vessel remodeling; cell differentiation | Protein binding; lipoprotein binding |
| NM_138455 | 2.64 | Collagen triple helix repeat containing 1 | Cell migration; positive regulation of protein binding | Protein binding; frizzled binding |
| NM_001511 | 2.44 | Chemokine (C-X-C motif) ligand 1 | Cell response to LPS; chemotaxis; G-protein coupled receptor signaling pathway; immune response; inflammatory response | Chemokine activity |
| NM_002994 | 2.42 | Chemokine (C-X-C motif) ligand 5 | Immune response; chemotaxis; signal transduction | Chemokine activity |
| NM_002993 | 3.00 | Chemokine (C-S-C motif) ligand 6 | Inflammatory, immune response; chemotaxis; signal transduction | Chemokine activity |
| NM_004887 | 2.43 | Chemokine (C-X-C motif) ligand 14 | Immune response; chemotaxis; signal transduction | Chemokine activity; cytokine activity |
| NM_198477 | 4.51 | Chemokine (C-X-C motif) ligand 17 | Chemotaxis; cell differentiation, angiogenesis | |
| NM_000772 | -2.89 | Cytochrome P450 family 2 subfamily C polypeptide 0 | Cellular amide metabolism; oxidative-reduction process; | Aromatase activity; oxidoreductase activity |
| NM_181745 | 4.30 | Cytohesin 3 | Establish epithelial cell polarity; inositol phosphate-mediated signaling | 1-phosphatidylinositol binding; |
| NM_014392 | 2.50 | DNA segment on chromosome 4 234 expressed sequence | Dopamine receptor signaling pathway | Dopamine receptor binding |
| NM_001017920 | -2.12 | Death-associated protein-like 1 | Apoptosis |  |
| NM_004942 | 10.47 | Defensin beta 4A (defensin 3) | Immune response; chemotaxis; G-protein  coupled receptor protein signaling pathway | |
| M_182643 | 2.39 | Dynein light chain,LCB-type 1 | Actin cytoskeleton organization; apoptosis; regulation of transcription | Enzyme binding |
| NM_021233 | -2.16 | Deoxyribonuclease II beta | DNA metabolic process | Endonuclease activity |
| NM_001937 | 2.86 | Dermatopontin | Cell adhesion, collagen fibril organization | |
| NM_005822 | 2.38 | Regulator of calcineurin 2 | Calcium-mediated signaling | Nucleotide binding; protein phosphatase 2B binding |
| NM_001397 | -2.95 | Endothelin converting enzyme 1 | Apoptosis; endothelin maturation; | Endopeptidase activity |
| NM_000115 | 3.55 | Endothelin receptor type B | Activation phospholipase C (G-protein coupled receptor protein signaling coupled to IP3 2nd messenger) | Endothelin-B receptor activity; G-protein coupled receptor activity |
| NR_027068 | 10.83 | EF-hand calcium binding domain 10 | Signal transduction; Regulation of phosphorylation | Calcium ion binding; cAMP-dependent protein kinase regulator activity |
| NM_004105 | 3.64 | EGF containing fibulin-like extracellular matrix protein 1 | Epidermal growth facor receptor signaling pathway; regualtion of transcription, DNA-dependent | Calcium ion binding; epidermal growth factor-activated receptor activity |
| NM_018100 | 2.20 | EF-hand domain (C-terminal) containing 1 | Apoptosis-positive regulation | Calcium ion binding |
| NM_001968 | 2.07 | eukaryotic translation initiation factor 4E | Cytokine-mediated signaling pathway; | Translation initiation factor activity |
| NM_022726 | -2.13 | ELOVL fatty acid elongase 4 | Metabolic process - cellular lipid | Transferase activity |
| NM_005232 | -2.55 | EPH receptor A1 | Activation of rho GTPase activity; ephrin receptor signaling pathway | ATP binding; protein kinase activity |
| NM_004447 | 2.88 | Epidermal growth factor receptor pathway substrate 8 | Epidermal growth factor receptor signaling; cell proliferation | Protein binding |
| NM_017697 | 5.09 | Epithelial splicing regulatory protein 1 | Regulation of mRNA splicing | mRNA binding |
| NM_004101 | 2.70 | Coagulation factor II (thrombin) receptor-like 2 | G-protein coupled receptor signaling; signal transduction | G-protein coupled receptor activity; signal transducer activity |
| NM_001080396 | 2.39 | Family with sequence  similarity 155, member A | | Binding |
| NM_000138 | 2.35 | Fibrillin 1 | Sequestering of TGF-β in extracellular matrix | Calcium ion binding; extracellular matrix structure constituent |
| NM_178820 | -2.37 | F-box protein 27 | Protein catabolism | Glycoprotein binding |
| NM_052939 | -2.09 | Fc receptor-like 3 |  | Receptor activity |
| NM_000508 | 3.02 | Fibrinogen alpha chain | Signal transduction; Platelet activation | Receptor binding; Protein binding |
| NM_004751 | 3.69 | Glucosaminyl (N-acetyl) transferase 3, mucin type | Immune response (Ig production in mucosal tissues) | Transferase activity (glycosyl groups) |
| NM_145740 | 8.54 | Gluthatione S-transferase alpha 1 | Metabolic process – glutathione | Glutathione transferase activity |
| NM_000847 | 2.91 | Glutathione-S-transferase alpha 3 | Metabolic process – glutathione | Glutathione transferase activity |
| NM_021957 | 2.78 | Glycogen synthase 2 | Metabolic process - carbohydrate | catalytic activity, glycogen synthase |
| NM_181353 | 2.11 | Inhibitor of DNA binding 1, dominant negative helix-loop-helix protein | Apoptosis; angiogenesis, Collagen metabolic process, Regulation of MAPK cascade | Protein binding; transcription factor activity |
| NM_000589 | -3.10 | Interleukin-4 | Inflammatory, immune response; chemotaxis, B cell activation | Cytokine; protein binding |
| NM_000584 | 5.13 | Interleukin-8 | Inflammatory, immune response, chemotaxis; G-protein coupled receptor protein signaling pathway | Chemokine; protein binding |
| NM_002187 | -2.44 | Interleukin-12B, p40) | Inflammatory, immune response, cell response to IFN-γ, LPS | Cytokine; protein binding |
| NM_020525 | 5.00 | Interleukin-22 | Inflammatory, acute phase response; cell-cell signaling | Cytokine; protein binding |
| NM_024726 | 2.97 | IQ motif containing with AAA domain 1 | Metabolic process | ATP binding; nucleotide binding |
| NM_014217 | 3.36 | Potassium channel, subfamily K, member 2 | G-protein coupled receptor signaling pathway | Ion channel activity; potassium channel activity |
| NM_013289 | -2.65 | Killer cell immunoglobulin-like receptor, three domains, long cytoplasmic tail, 1 | Immune response | MHC class I receptor activity |
| NM_130446 | -2.08 | Kelch-like g (Drosophila) | Immune response: B cell receptor signaling  Pathway | |
| NM_002257 | 2.26 | Kallikrein | Inflammatory response; apoptosis; cell proliferation | Endopeptidase activity |
| NM_006121 | -2.77 | Keratin 1 | Complement activation, lectin pathway; response to oxidative stress | Protein binding; receptor activity |
| NM_000423 | -2.51 | Keratin 2 | Epidermis development; keratin proliferation | structural constituent of cytoskeleton |
| NM_000224 | 3.15 | Keratin 18 | Negative regulation of apoptosis; CFTR protein transport | Protein binding; structural molecule activity |
| NM_002276 | 2.09 | Keratin 19 | Cell differentiation | Protein binding; structural component of cytoskeleton |
| NM_181537 | -2.16 | Keratin 27 | Hair follicle morphogenesis | Structural molecule activity |
| NM_002273 | 3.25 | Keratin 8 | Apoptosis; Cell morphogenesis; Cytoskeleton organization; TNF-mediated signaling pathway | Protein binding; structural molecular activity |
| NM_005558 | -2.41 | Ladinin 1 | Epithelial structure | Enzyme binding; transcription factor binding |
| NM_001290 | 2.15 | LIM domain binding 2 | Epithelial structure; positive regulation of transcription from RNA polymerase II promoter | Enzyme binding; transcription factor binding |
| NM_002308 | -3.04 | Lectin, galactoside-binding, soluble 9 | positive regulation of I-kappaB kinase/NF-κB cascade | Signal transduction, sugar binding |
| NM_012276 | 4.07 | Leukocyte  immunoglobulin-like  receptor, subfamily A member 4 | | Receptor activity |
| NM_181879 | 2.59 | Leukocyte  immunoglobulin-like  receptor, subfamily A member 4 | | Receptor activity |
| NM_170707 | 2.16 | lamin A/C | Apoptosis | Protein binding; structural molecule activity |
| NM_001080434 | -3.97 | Lemur tyrosine kinase 3 | Negative regulation of phosphatase activity | ATP binding; kinase activity,; nucleotide binding |
| NM_002317 | 2.28 | Lysyl oxidase | Oxidation-reduction process; protein deamination | Oxidoreductase activity |
| NM_005576 | 2.53 | Lysyl oxidase-like 1 | Oxidation-reduction process; protein deamination | Oxidoreductase activity |
| NM_032211 | 2.10 | Lysyl oxidase-like 4 | Oxidate-reduction process | Oxidoreductase activity |
| NM_015236 | 3.18 | Latrophilin 3 | G-protein coupled receptor signaling pathway; signal transduction | Signal transduction |
| NM_005824 | 2.57 | Leucine rich repeat containing 17 | Negative regulation of osteoclast  Differentiation | |
| NM_002345 | 3.44 | Lumican | Collagen fibril organization | Extracellular matrix structural constituent |
| NM_003926 | 3.92 | Methyl-CpG binding domain protein 3 | Transcription | Chromatin binding, DNA binding, protein binding |
| NM_001012755 | -2.28 | Mitochondrial carrier triple repeat 6 | Transport |  |
| NM_002395 | 2.06 | Malic enzyme 1, NADP(+)-dependent, cytosolic | Metabolic process - carbohydrate; oxidation-reduction process | ADP binding; oxidoreductase activity |
| NM_005241 | 3.63 | MDS1 and EVI1 complex locus | Apoptosis; inflammatory process; | DNA binding |
| NM_002402 | 2.23 | Epoxide hydrolase | Metabolic process; regulation of lipid storage | Hydrolase activity |
| NM_003480 | 3.53 | Microfibrillar associated protein 5 |  | Extracellular matrix constituent |
| NM_005511 | 2.81 | Melan-A |  | Protein binding |
| NM_002421 | 6.17 | Matrix metallopeptidase 1 | Proteolysis, collagen catabolism | Metalloendopeptidase activity; Calcium ion binding |
| NM_004994 | 2.49 | Matrix metallopeptidase 9 | Cell response to IL-1, TNF; hypoxia; LPS | Collagen binding; hydrolase activity |
| NM_007351 | 2.72 | Multimerin 1 | Cell adhesion; blood coagulation |  |
| NM_022746 | 2.01 | Mitochondrial amidoxime reducing component 1 | Nitrate metabolic process; oxidation-reduction process | Oxidoreductase activity |
| NM_001584 | -2.08 | Metallophosphoesterase domain containing 2 | Nervous system development | Hydrolase activity |
| NM_207243 | -2.38 | Mucin 19, oligomeric | Cellular protein metabolic process | P-P bond hydrolysis driven protein transmembrane transporter activity |
| NM_002465 | ­­2.04 | Myosin binding, structural constituent of cytoskeleton | Cell adhesion; blood coagulation | Structural constituent of cytoskeleton |
| NM_002471 | -2.25 | Myosin, heavy chain 6, cardiac muscle alpha | ATP catabolic process; BMP signaling pathway, canonical Wnt receptor signaling pathway | Actin binding-ATPase activity |
| NM_013451 | 2.29 | Myoferlin | Regulation of vascular endothelial growth factor receptor signaling pathway | Phospholipid binding |
| NM_152372 | 2.37 | Myomesin family, member 3 |  | Protein homodimerization activity |
| NM_021245 | 3.13 | Myozenin 1 | Myofibril assembly | FATZ binding |
| NM_022150 | 2.09 | Neuropeptide VF precursor | Neuropeptide signaling; negative regulation of gonadotropin secretion | G-protein coupled receptor acivity |
| NM_004801 | 2.16 | Neurexin 1 | Cell adhesion, | Acetylcholine receptor binding; Cell adhesion molecule binding |
| NM_014057 | 3.21 | Osteoglycin | Negative regulation of smooth muscle cell proliferation | Growth factor activity; protein binding |
| NM_005014 | 3.40 | Osteomodulin | Cell adhesion; regulation of bone mineralization | |
| NM_017734 | 3.66 | Palmdelphin | Regulation of cell shape | Protein binding |
| NM_133367 | 2.40 | Progestin and adipoQ receptor family member VIII | Cell differentiation | Lipid binding; receptor activity; steroid binding |
| NM_000926 | 2.09 | Progesterone receptor | Cell-cell signaling; epithelial cell maturation; gene expression | DNA binding; enzyme binding |
| NM_002653 | 3.05 | Paired-like homeodomain 1 | Regulation of transcription, DNA-dependent | DNA binding, protein binding transcription factor acivity |
| NM_000304 | 2.12 | Peripheral myelin protein 22 | Cell cycle; neg regulation of cell proliferation | |
| NM_002728 | -3.72 | Proteoglycan 2 | Immune response; defense to bacteria | Binding; sugar binding |
| NM_006093 | -3.78 | Proteoglycan 3 | Immmune response; pos reg IL-8, histamine, leukotriene biosynthesis | Sugar binding |
| NM_005807 | 6.42 | Proteoglycan 4 | Immune response; receptor-mediated endocytosis | Polysaccharide binding |
| NM_004878 | 2.09 | Prostaglandin E synthase | Inflammatory response; fatty acid biosynthesis; response to LPS, retinoic acid; signal transduction | Synthase activity |
| NM_000963 | 2.20 | Prostaglandin-endoperoxide  synthase 2 (prostaglandin G/H  syntase and cyclooxygenase) | | |
| NM_002852 | 4.33 | Pentraxin 3, long | Inflammatory response, positive regulation of NO biosynthesis; phagocytosis | Zymosan binding |
| NM_001008749 | -2.58 | RAB 19, member RAS oncogene family | Protein transport; small GTPase mediated signal transduction | GTP binding |
| NM_006266 | 2.60 | Ral guanine nucleotide dissociation stimulator | Ras protein signal transduction; regulation of small GTPase mediated signal transduction; signal transduction | Protein binding; small GTPase regulator activity |
| NM_006744 | 2.23 | Retinol binding protein 4, plasma | Positive regulation of Ig secretion; lung development | Protein binding; transporter activity |
| NM_012102 | -2.00 | Arginine-glutamic acid dipeptide (RE) repeats | Chromatin remodeling; regulation of transcription | DNA binding; protein binding |
| NM_002927 | -2.54 | Regulator of G-protein signaling 13 | G-protein coupled receptor signaling pathway; neg regulation of signal transduction | GTPase activator activity; protein binding |
| NM_001029875 | 3.11 | Regulator of G-protein signaling 7 binding protein | G-protein coupled receptor signaling pathway; negative regulation of signal transduction | Protein binding |
| NM_173642 | 3.20 | Ribosomal modification protein rimK-like family member A | Protein modification process | ATP binding; ligase activity |
| NM_020914 | -2.69 | Ring finger protein 213 | Protein auto-ubiquitination | ATPase activity; ubiquitin-protein ligase activity |
| NM_017578 | 4.09 | rhophilin associated tail protein 1 |  | cAMP-dependent protein kinase regulator activity |
| NM_031916 | 2.60 | Rhophilin associated tail protein 1-like | regulation of phosphorylation; signal transduction; ciliary motility | cAMP-dependent protein kinase regulator activity |
| NM_199161 | 7.36 | Serum amyloid A1 | Inflammatory, acute phase response; chemotaxis (PMN, mac, LO0, reg IL-1) | G-protein-coupled receptor binding |
| NM_030754 | 4.81 | Serum amyloid A2 | Acute phase response, chemotaxis (lymphocyte) | G-protein coupled receptor binding |
| NM_000541 | 2.25 | S-antigen | Cell surface receptor signaling pathway | phosphoprotein binding |
| NM_015265 | 2.60 | SATB homeobox | Neg reg transcripton from RNA polymerase II promoter | Chromatin binding; |
| NM_016240 | 2.02 | Scavenger receptor, transmembrane receptor | Response to oxidative stress | Receptor binding |
| NM_003357 | 3.82 | Secretoglobulin family 1A member 1 | Negative regulation of IL-4,-5, -13 production; IFN-γ regulation in inflammatory response; response to LPS | Phospholipase A2 inhibitor activity |
| NM_054023 | 3.41 | Secretoglobulin family 3A member 2 |  | Protein binding |
| NM_003005 | 3.14 | Selectin P | Positive regulation of leukocyte migration; inflammatory response, LPS | LPS binding; protein, sugar binding |
| NM_006379 | 2.85 | Sema domain, immunoglobulin domain short basic domain secreted, (semaphorin) 3C | Immune response; pulmonary myocardium development | Receptor activity |
| NM_001080537 | 6.68 | Sentan | Cilia component, respiratory epithelium | Calcium ion binding |
| NM_003018 | 5.16 | Surfactant protein C | Respiratory gas exchange (surface tension) | Protein homodimerization activity |
| NM_152527 | 2.22 | Solute carrier family 16, member 14 | Transmembrane transport | Symporter activity |
| NM_003054 | -2.29 | Solute carrier family 18 member 2 | Regulation of neurotransmitter transport, secretion | Amine transmembrane transporter |
| NM_003064 | 2.76 | Secretory leukocyte peptidase inhibitor |  | Endopeptidase inhibitor activity |
| NM_005905 | 2.30 | SMAD family member 9 | TGF beta receptor signaling pathway; | Protein binding; transforming growth factor beta receptor |
| NM_022138 | 2.18 | SPARC related modular calcium binding 2 | Extracellular matrix organization; signal transduction | Glycosaminoglycan binding |
| NM_199262 | 3.08 | Sp6 transcription factor | Positive regulation of cell proliferation; regulation of transcription | DNA binding |
| NM_206996 | 4.29 | Sperm associated antigen 17 |  | Protein binding; transforming growth factor beta receptor |
| NM_138796 | 2.20 | Spermatogenesis associated 17 |  | Calmodulin binding |
| NM_012449 | 2.07 | Six transmitter epithelial antigen of the prostate 1 | electron transport chain; oxidation-reduction process | Channel activity; oxidoreductase activity; transporter activity |
| NM_031414 | -2.29 | Serine/threonine kinase 31 | Protein phosphorylation | ATP binding; kinase activity, protein kinase activity |
| NM_015551 | 2.95 | Sushi domain containing 5 | Cell adhesion | Binding hyaluronic acid |
| NM_206862 | 2.50 | Transforming acidic coiled-coil containing protein 2 | Regulation of microtubule-based process | Nuclear hormone receptor binding; protein specific binding |
| NM_153046 | -2.27 | Tudor domain containing 9 | ATP catabolic process; cell differentiation | ATP binding; hydrolase activity |
| NM_003225 | 4.19 | Trefoil factor 1 | Metabolic process - carbohydrates; response to stress, peptide hormone stimulus | Growth factor activity |
| NM_007112 | 2.12 | Thrombospondin 3 | Cell adhesion; cell-matrix adhesion | Calcium ion binding; structural molecule activity |
| NM_006288 | 2.31 | Thy-1 cell surface antigen | Negative regulation of T-cell receptor signaling pathway; cell-cell adhesion, mast cell activation, negative regulation of cell migration | Enzyme binding; integrin binding |
| NM_001079668 | 2.78 | NK2 homeobox 1 | Negative regulation of transcription; TGF-β1 signaling; LPS | Transcription factor activity |
| NM_007115 | 5.44 | Tumor necrosis factor, alpha-induced protein 6 | Inflammatory response; signal transduction | Hyaluronic acid binding |
| NM_006034 | -3.06 | Tumor protein p53 inducible protein 11 | Negative regulation of cell proliferation;  response to stress | |
| NM_030773 | -2.07 | Tubuline beta 1 class VI | De novo posttranslational protein folding; GTP catabolic process | GTPase activity; structural molecule activity |
| NM_178130 | 2.74 | NME family member 9 | Cell redox homeostasis, GTP biosynthetic process | ATP binding; nucleoside diphosphate kinase activity |
| NM_173856 | -2.00 | Vomeronasal 1 receptor 2 | G-protein coupled receptor signaling pathway; response to pheromone | Signal transducer activity |
| NM_007128 | -12.12 | Pre-B lymphocyte 1 | immune response | protein binding; antigen binding |
| NM_030761 | -2.04 | Wingless type MMTV integration site family, member 4 | Canonical WNT receptor signaling pathway; mesenchymal to epithelial transition; | extracellular matrix structural constituent; G-protein coupled receptor biding |
| NM_015472 | 2.18 | WW domain containing transcription regulator 1 | Cilium morphogenesis; neg reg canonical WNT receptor signaling pathway |  |
| NM_001136571 | -2.11 | Zygote arrest 1-like | sodium ion transport | protein binding |
| NM_181877 | -2.21 | Zinc finger and SCAN domain containing 2 | cell differentiation; regulaton of transcription | DNA binding; |
| NM_198493 | 2.30 | Ankyrin repeat domain 45 |  |  |
| NM_025145 | 3.49 | WD repeat domain 96 |  |  |
| NM_032930 | 3.56 | Chromosome 11 ORF 70 |  |  |
| NM_030572 | 2.91 | Chromosome 12 ORF 39 |  |  |
| NM_152325 | 3.58 | Testis expressed 26 |  |  |
| NM_182508 | 6.19 | Family with sequence  similarity 216, member B | |  |
| NM_032849 | 3.48 | Chromosome 13 ORF 33 |  |  |
| NM_001013625 | 2.93 | Chromosome 1 ORF 192 |  |  |
| NM_001145474 | 2.11 | ATPAF1 antisense RNA 1 |  |  |
| NM_016613 | 2.01 | Family with sequence  similarity 198, member B | |  |
| NM_152770 | 3.17 | Chromosome 4 ORF 22 |  |  |
| NM_206966 | 2.73 | Chromosome 5 ORF 46 |  |  |
| NM_032744 | -2.71 | Androgen-dependent TFPI-regulating protein |  |  |
| NM_024033 | -2.48 | Chromosome 7 ORF 49 |  |  |
| NM_198469 | 3.95 | MORN repeat containing 5 |  |  |
| NM_018956 | 3.62 | Chromosome 9 ORF 9 |  |  |
| NM_017863 | -3.23 | Chromosome X ORF 48 |  |  |
| NM_020893 | 3.86 | Chromosome 9 ORF 174 |  |  |
| NM_016438 | 2.31 | HIG1 hypoxia inducible  domain family, member 1B | |  |
| NM_032263 | 2.82 | IQ motif containing G |  |  |
| NM_001012973 | 2.68 | Placenta-specific 9 |  |  |
| NM_181774 | 2.21 | Solute carrier family 36 member 3 |  |  |
| NM_015204 | 2.84 | Thrombospondin type I domain containing 7A |  |  |
| NM_024780 | 2.92 | Transmembrane channel-like 5 |  |  |
| NM_014220 | 2.76 | Transmembrane protein 47 |  |  |
| NM_021202 | 3.36 | Tumor protein p53 inducible nuclear protein 2 |  |  |
| NM_018117 | -2.08 | WD repeat domain 11 |  |  |
| NM_145172 | 3.03 | WD repeat domain 63 |  |  |
| NM_144668 | 2.18 | WD repeat domain 66 |  |  |
| NM_178821 | 3.28 | WD repeat domain 69 |  |  |
