# Supplementary material for: Differential Gene Expression Profiles and Selected Cytokine Protein Analysis of Mediastinal Lymph Nodes of Horses with Chronic Recurrent Airway Obstruction (RAO) Support an Interleukin-17 Immune Response
Source: PLoS One. 2015 Nov 12;10(11):e0142622. doi: 10.1371/journal.pone.0142622 (PMC4642978; doi:10.1371/journal.pone.0142622)
Supplement: S3 Table — (DOCX) [file pone.0142622.s003.docx]

**Table S3. Fold-change (RAO:control) in expression of 10 genes measured by RT-PCR and microarray assays**

| **Gene** | | **RT-PCR** | **Microarray** |
| --- | --- | --- | --- |
| DEFB4A | Beta defensin 3 | ↑ 11.6 | ↑ 10.4 |
| CALCB | Calcitonin-related polypeptide beta | ↓ 1.3 | ↓ 3.2 |
| CD163 | Hemoglobin/haptoglobin receptor scavenger | ↑ 19.2 | ↑ 7.4 |
| CXCL1 | Chemokine (C-X-C motif) ligand 1 | ↑ 2.3 | ↑ 2.4 |
| CXCL6 | Chemokine (C-X-C motif) ligand 6 | ↑ 2.5 | ↑ 3.0 |
| IL8 | Interleukin-8 | ↑ 7.7 | ↑ 5.1 |
| PRG4 | Proteoglycan 4 | ↑ 13.4 | ↑ 6.4 |
| RELA | V-rel avian reticuloendotheliosis viral oncogene homolog A (NFκB p65) | ↑ 1.3 | ↑ 1.4 |
| TP53i11 | Tumor protein p53 inducible protein 11 | ↓ 1.2 | ↓ 3.1 |
| IL17 | Interleukin 17 | ↑ 1.1 | ↑ 1.1 |
